# Is There a Relationship Between Grip Strength and Injuries in Professional Baseball Players? Letter to the Editor

**DOI:** 10.1177/23259671251342815

**Published:** 2025-06-30

**Authors:** Justin M. Losciale, Chad E. Cook, Ava Sina, Patrick Ward

Dear Editor:

As researchers who share a similar passion to Erickson and colleagues, we feel compelled to comment on their recently published paper titled, “Is There a Relationship Between Grip Strength and Injuries in Professional Baseball Players?”^6^ In their work, they found no differences in mean grip strength for pitchers who did and did not seek medical attention for arm complaints during 1 season.^6^ While we appreciate their findings, we’d like to highlight a number of additional considerations required when designing a study to understand the relationship between grip strength on injury risk.

The authors suggest the study is a case-control design. Case-control study designs are a retrospective analysis where patients with the disease of interest (ie, cases) are selected into the study and then compared with select patients who do not have the disease (ie, the controls) to understand the exposure pattern for the source population.^16^ Traditional case-control sampling strategies cannot ascertain rates, risk, or probabilities because of the independent retrospective sampling of cases and controls based on the disease of interest.^13^ Since the authors prospectively evaluated grip over the course of the season in a single Major League Baseball (MLB) organization, this study is not a case-control but a prospective cohort design. In other words, all pitchers were included before outcome status (ie, sustaining an injury), selected based on exposure (ie, pitching in the single MLB organization), and were followed over time.^1^ This is relevant, as prospective cohorts also allow calculations of incidence rates, risk, and probabilities as they sample based on exposure from the entire population (not on outcome such as in a case-control),^3^ which in this case was a single MLB organization.

The authors use a unique definition of injury. To compare groups, the authors utilized an outcome-based definition of “any shoulder or elbow problem that caused the player to be evaluated by the medical staff and have a treatment regimen implemented.”^6^ This is a definition of seeking medical attention, and is a pertinent outcome to assess.^11^ While not a standard outcome measure for injury, providing this definition can permit researchers to calculate the use of medical resources, however it does not allow inferences concerning an athlete’s ability to participate in current or future practices or games. Time-loss outcomes allow for injury severity and burden calculations.^2^ We want to emphasize that an outcome of “seeking medical attention” may be used, but one should consider incorporating an adjoining time-loss outcome definition.

The time between measurement and injury investigated in this study is likely not sensitive to changes local to the injury event. One of the authors’ stated goals was to compare mean grip strength measured across the entire season after pitching appearances between pitchers who received medical attention (ie, their definition of injury) and those who did not.^6^ To identify athletes of concern, an entire season’s worth of grip strength measures would need to be collected first. This makes data collection infeasible and does not allow clinicians to identify players at risk of injury during the preseason or during the season.

Time-varying effects of grip strength need to be accounted for throughout the course of the season. Time-varying effects are when a value changes over time, which then changes its association with the outcome.^12,15^ It is well-established that physical performance parameters such as strength,^9^ power,^8,14^ and neuromuscular function^5,7^ can vary within an individual or groups over the course of an athletic cycle (eg, between pitching starts) or throughout the course of the season. This is what we assume the authors intended to explore when they mentioned exploring an association between fatigue and injury. Including an interaction with time—and further exploration of the nonlinear association between time, grip strength, number of pitch exposures per game, and the outcome of interest—would enable us to understand the potential time-varying effects and draw an inference to this stated goal.

Intrapitcher relationships between grip strength measures need to be accounted for in the analyses. There is a strong similarity (ie, correlation) between serial grip measures for each individual pitcher.^4^ Accounting for this correlational structure between repeated measures allows grip strength patterns to be assessed in relation to trajectories in reaching the outcome.^10^ For example, there may be a distinct difference in preseason grip strength measures between pitchers who will and will not experience an arm injury during the season, or the rate of change in grip strength may be a determining factor in arm injury risk during the season.^10^ Accounting for the intrapitcher relationship between serial grip strength measurements can allow clinicians to understand these important clinical questions.

While the questions Erickson et al^6^ set out to ask are significant, the methodology used does not allow inference toward those questions in this population or others. We hope to foster an open discussion around the multiple methodological considerations that should be addressed to answer the stated questions, in order to raise our professional awareness regarding these common concerns. We hope this discussion highlights the need for collaborating with epidemiological and statistical experts to create well-designed studies that can precisely evaluate injury prevention questions, to ultimately translate scientific results from “bench to bedside.”


Justin M. Losciale, PT, DPT, PhD
*Salt Lake City, UT, USA*
Scot Morrison, PT, DPT, OCS, CSCS
*Verona, Italy*
Chad E. Cook, PT, PhD, MBA
*Durham, NC, USA*
Ava Sina, BA
*El Paso, TX, USA*
Patrick Ward, PhD
*Seattle, WA, USA*

